# Toward a Symbiotic Perspective on Public Health: Recognizing the Ambivalence of Microbes in the Anthropocene

**DOI:** 10.3390/microorganisms8050746

**Published:** 2020-05-16

**Authors:** Salla Sariola, Scott F. Gilbert

**Affiliations:** 1 Faculty of Social Sciences, Sociology, University of Helsinki, 00014 Helsinki, Finland; salla.sariola@helsinki.fi; 2 Department of Biology, Swarthmore College, Swarthmore, PA 19081, USA

**Keywords:** microbial evolution, public health, symbiosis, drug resistance, hologenome, AMR, allergy, anthropocene, plantationocene

## Abstract

Microbes evolve in complex environments that are often fashioned, in part, by human desires. In a global perspective, public health has played major roles in structuring how microbes are perceived, cultivated, and destroyed. The germ theory of disease cast microbes as enemies of the body and the body politic. Antibiotics have altered microbial development by providing stringent natural selection on bacterial species, and this has led to the formation of antibiotic-resistant bacterial strains. Public health perspectives such as “Precision Public Health” and “One Health” have recently been proposed to further manage microbial populations. However, neither of these take into account the symbiotic relationships that exist between bacterial species and between bacteria, viruses, and their eukaryotic hosts. We propose a perspective on public health that recognizes microbial evolution through symbiotic associations (the hologenome theory) and through lateral gene transfer. This perspective has the advantage of including both the pathogenic and beneficial interactions of humans with bacteria, as well as combining the outlook of the “One Health” model with the genomic methodologies utilized in the “Precision Public Health” model. In the Anthropocene, the conditions for microbial evolution have been altered by human interventions, and public health initiatives must recognize both the beneficial (indeed, necessary) interactions of microbes with their hosts as well as their pathogenic interactions.

## 1. Introduction

The Anthropocene marks the end of a period characterized by the human triumph of nature, and nowhere is this more prominent than in public health. The heroic era of microbiology made Pasteur, Koch, Lister, and Fleming household names, and few have done more for humanity [1,2,3]. The lifespan of a newborn Parisian was 45 years in 1900; and a century later, a newborn Parisian could expect to live another 80 years [4]. Much of this increase in life expectancy (as well as the expectation to be healthy) has been due to the ability of public health measures—proper sanitation, food surveillance, antisepsis, and anti-viral vaccination—to remove humans from sources of microbial infection.

However, our relationship with microbes and their evolution has changed dramatically since the discovery of antibiotics, and the conquest of polio and other endemic viruses in the 1950s and 1960s. The first part of our changing relationship to microbes involves the Western world’s manufacturing an environment that is increasingly sterile and characterized by the removal of unplanned and unplannable nature [5,6]. Indeed, nature is no longer our “state of nature.” Rather, we have continually separated ourselves from nature, separating the “who” of human lives from the “what” of other lives. To be modern is to be removed from nature [7,8]. More-than-human parts of the world are becoming domesticated and industrially managed. Biomes are shrinking to be replaced by cities and managed farms [9]. Nature is “dead” in that it is no longer partaking of the sublime, that which is always dangerous, powerful, majestic and outside us [10,11]. We now know the earth as a plantation, where hierarchical and rational management practices enlist coercive migration and reduced biodiversity to regulate the planetary resources to feed the growing human population. The opportunities for rapid global travel and the concentration of humans into new urban areas in the Anthropocene have converted what would have been local outbreaks into planetary pandemics. Emerging infectious diseases have been exacerbated by land clearing and habitat fragmentation [12], and there is speculation that the current outbreak of SARS-CoV2 is linked to the loss of native forests [13] as well as trafficking of wild life [14,15,16]. Dominion over and extractive harnessing of humans and other species for financial profit has been called the Plantationocene [17,18]. Like animals, even microbes are “harnessed” to support human needs [19].

The second part of our changing relationship to microbes and their evolution is that the microbes, themselves, have changed. Indeed, part of that fear of microbes is justified, as the Anthropocene has created new conditions for microbial expansion [1,20]. This essay is being written in the first months of the social upheaval threatened by SARS-CoV2, a virus that did not exist during the times of Pasteur and Koch [15,21]. Moreover, our unregulated use of antibiotics has created microbes that have evolved resistance to our normally prescribed anti-microbial drugs. Prior to WWII, hardly any bacteria harbored genes that could degrade antibiotics. However, selection pressure from antibiotics, disinfectants, and heavy metals created the conditions where rare variants containing drug-resistant genes could selectively propagate [22,23,24,25]. This drug resistance has spread more rapidly than scientists had predicted, because it was only in the 21st century that biologists began to recognize the importance of lateral gene transfer (also called horizontal gene transfer). Bacteria can acquire genes from their neighbors as well as acquiring genes from their parent. Indeed, the *E. coli* in our guts obtain more variation from lateral gene transfer than they do from mutation [26]. Drug-resistant infections now cause 700,000 deaths each year, and the World Health Organization predicts that without drastic interventions, drug-resistant pathogens might be responsible for 10 million deaths globally per year by 2050 [27].

The third change in our relationship with microbes and their evolution is the belated recognition of mutualistic symbiosis. Medicine and public health have focused almost exclusively on competitive parasitic symbioses, on the pathogenicity of microbes. However, symbiosis is performed in two major modes: mutualism (where both parties benefit) and parasitism (where one party benefits at the expense of the other). As we will see, symbiotic bacteria are critical for our bodies’ health and maintenance. They are essential partners in allowing our immune system, our endocrine system, and our nervous system to operate [28,29]. It is worth mentioning that mutualism is not only the prerogative of bacteria and archaea. Despite the pathogenic ravages brought about by Ebola virus and SARS-CoV-2, viruses can also be in important mutualist relationships with their hosts [30,31,32,33].

We thus arrive at the powerful ambivalence of microbes in the Anthropocene. On the one hand, microbes are defined as the foremost enemy of humans. In our failed attempts to eliminate microbes from our lives, the Anthropocene has created the conditions for recombinant viruses and antibiotic-resistant bacteria. On the other hand, we have recognized that microbes are part of our very being. Our health depends on the symbiotic bacteria that helps build and maintain our healthy bodies. The body’s immune system has evolved to recognize the difference between benign and potentially pathogenic bacteria.

Thus, while some anthropogenic conditions have caused certain mutualistic microbes to be endangered or exchanged, other anthropogenic conditions have enabled certain pernicious microbes to increase both in prevalence and toxicity. This paradox can be resolved by recognizing two critically important characteristics of microbes that had been marginalized by the biomedical community prior to the 21st century: (1) the integration of microbes into the physiology, anatomy, development, and immune systems of plants and animals and (2) the microbes’ ability to transfer DNA horizontally from organism to organism. This article attempts to map out a holobiont perspective to public health.

### 1.1. Anthropocene Public Health Initiatives

It is important to determine how these new views of microbial evolution—lateral gene transfer and mutualistic symbiosis—might be integrated into public health initiatives. It seems that present initiatives ignore or marginalize these phenomenon and that public health might be served better if they were made more central. Two pertinent public health paradigms that have received much publicity in recent years are the “Precision Public Health” (PPH) paradigm and the “One Health” (OH) paradigm. Neither of these appear to take seriously our new appreciation of microbial evolution.

### 1.2. Precision Public Health

Precision Public Health (PPH) is the application of genomics technology for population health benefits [34,35], and it is the attempt to make public health into a genomic science. PPH began in 1997, when the Office of Public Health Genomics of the CDC was formed to “transform” population health care into a genomic science “by identifying, evaluating, and implementing evidence-based genomics practices to prevent and control the country’s leading chronic, infectious, environmental, and occupational diseases” [36]. PPH claims that it would be able to analyze one’s genome and then prescribe the appropriate drugs and dietary regimens.

However, the original promises that genomic science would find common alleles for common diseases were not fulfilled [37,38]. Genome-wide association studies (GWAS) for cardiovascular disease showed that that genes played a negligible role in predicting heart attacks and that human genetic variation accounted for roughly 3% of the variation in blood pressure [39]. Moreover, the prediction that a patient would have a heart attack was better made by the number of pushups a patient could do than by genomic analyses [40]. The genes thought to be associated with depression were “lost” when large trials were done [41]; and deficiencies of the gut microbiome may provide a better account of causation [42].

Worse, for any genomic model of public health, was when the genome of the founder of the Human Genome Project, James Watson, was analyzed. His DNA sequences predicted him to be deaf, blind, growth retarded, and mentally deficient [43,44]. Genes work differently in different people. “Phenotypic heterogeneity,” wherein the same mutant allele causes different phenotypes in different individuals carrying it, is a well-known phenomenon in medical genetics [45,46] and a gene that is “normal” in one generation can cause disease in another [47,48,49].

Nevertheless, the PPH got a shot in the arm (to use an old public health metaphor) by the “All of Us” project begun at the USA’s National Institutes of Health [50]. Its website proclaims this to be a big genome, big data approach to public health, whereby “taking into account individual differences in lifestyle, environment, and biology, researchers will uncover paths toward delivering precision medicine...” PPH is getting a shot in the other arm from pharmacogenomics, the study of how responses to drugs are influenced by the genetic makeup of the person receiving the drug. According to Kapoor et al. [51] pharmacogenomics, is “one of the cornerstones of precision medicine” and furthermore, is a “significant innovation in health care that possesses the potential to change the paradigm in the practice of medicine, not solely in the way drugs are prescribed, but also in the way drugs are discovered and developed.” Indeed, precision pharmacogenetics is being touted as a paradigm for third-world health care [52].

However, this population-centered model of genomic healthcare delivery has been criticized [37] as being a salvage attempt to rescue something of value from the numerous extremely expensive genome projects that had been the scientific rage of the late 20th and early 21st centuries. Reardon and others [37,53,54] claim PPH is most likely dangerous fantasy, exacerbating global economic differences, taking the “public” out of “public health,” and shifting responsibility for health onto the individual citizen [55,56]. PPH has also been criticized for not recognizing the contributions of the symbiotic microbial genomes [38]. Symbiotic relationships with microbes, as will be discussed below, provide essential metabolic pathways for phenotype production (including those for drug metabolism) and over ten-fold the number of different genes than the zygote-derived genome.

### 1.3. One Health

Whereas Precision Public Health works from one privileged level—the genome—up to humans and human communities, the One Health paradigm is consciously interdisciplinary and multi-species—it attempts to envision people, animals, and environments as partners in each other’s health on several levels [57,58]. As Gibbs [59] (p. 49) notes, “One Health is the collaborative effort of multiple disciplines—working locally, nationally, and globally—to attain optimal health for people, animals, and our environment.” The One Health paradigm, according to Friese and Nuyts, provides a theoretical basis for research involving nonhumans in public health and used to re-organize relationships between human medicine and animal veterinary medicine so that these two fields communicate in both knowledge and practice [60].

With contributions from such disciplines as ecosystem services and soil microbiology, One Health approach also recognizes the role of environments and ecologies in how human and animal health is shaped. These contributions see ecosystems (including symbiotic microbiomes) as providing economic infrastructure benefits that can be calculated as part of any managed change to the environment [61,62,63]. However, overall, the implementation of One Health is still fixed on protecting humans from zoonotic infections [60]. Indeed, the CDC, WHO, AMA, and AVMA websites stress zoonoses and the fact that most infectious diseases are spread by animals. The environment gets short shrift in these sites, and this deficiency has not gone unnoticed. Numerous investigators have documented that the environment does not receive attention or funding in most One Health networks [64,65,66,67].

Thus, the three components of the One Health model are not equal, and the framework is still used to prioritize protection of humans from zoonotic diseases. While the importance of this goal is made obvious in this coronavirus-infused decade, the anthropogenic deterioration of the environment by humans—such as mountain-top coal removal, anthropogenic deforestation, soil microbial deterioration, and reef depletion— are crucial in and of themselves as well as can have enormous effects on public health and do not appear on the One Health agenda. Only recently have there been calls to put microbes and global climate change under the One Health umbrella [68,69]. Some of these initiatives have come under the Planetary Health [70], which emphasizes how critical such ecological perspectives are for human health. The Planetary Health perspective, however, concentrates on the important issues of politics and economics of global health care in the Anthropocene, but it does not address the issues the changes in how we perceive microbes.

### 1.4. Hologenome Theory: A Symbiosis of Environmental and Molecular Health 

In contrast to the Precision Public Health and One Health paradigms, a recently proposed theory holds that microbes such as bacteria are primarily beneficial symbionts of the human body, and their presence is both expected and necessary for normal human health. While pathogenic microbes can cause enormous damage, they are a distinct minority, and public health needs to recognize the other arm of symbiosis—mutualism. This approach, which could revitalize the community-based One Health perspective to public health by using the techniques of the molecularly based PPH model, is based on the hologenome theory [71]. This model has recently received support by private funding, most notably from the Bill and Melinda Gates Foundation [72,73].

The hologenome theory [74,75] recasts the individual animal or plant (and other multicellular organisms) as a consortium (“holobiont”)—the host plus all its symbiotic microbes. During the past two decades, advances in microbiome research have clearly shown that most animals cannot normally develop, function, or reproduce without the vast numbers of microorganisms that inhabit their bodies [28,76]. Microbes are essential for normal animal development and physiological functioning. For instance, bacteria acquired at birth from the female reproductive tract are critical to the construction of the gut capillaries and epithelia in several vertebrates [77,78,79,80,81] as well as being critical for the normal development of the vertebrate enteric and cerebral nervous systems [82,83,84].

Pediatric geneticist Barton Childs [85] postulated that each person’s genetic endowment constitutes a “biochemical individuality” conferred upon us by our genes. Patterson and Turnbaugh [86] have used the same term to designate the properties of the “hologenome”—the genes we inherit from our parents and our microbes, our germ cells and our germs. While we inherit some 22,000 genes from our parents, we inherit about 8 million different genes from our parents’ bacteria [87]. Indeed, in some instances, the gut microbiome appears to be critical in drug metabolism. Digoxin, cyclophosphamide, and numerous other drugs are each metabolized differently by different populations of microorganisms [88,89,90], giving each person an assortment of genes (and drug-metabolizing phenotypes) that can change with each meal [91]. 

Even the human immune system, so critical in public health, is a holobiont property, and not merely the agency of the host [92,93,94,95,96]. Microbes enter into the body at birth, prior to the maturation of the immune system, and they induce the formation of lymphoid tissues [97,98,99]. Moreover, these lifelong immune activities are well-regulated only in the continuous presence of microbes, which in turn, constantly regulate the microbes that can stay with the animal [100,101,102,103,104,105,106]. The immune system is a continuously co-constructed property of the holobiont. 

Holobiont public health recognizes that microbes may be pathogenic or beneficial, and that deficiencies in bacteria can cause developmental, immunological, cognitive, and physiological ailments. For instance, kwashiokor, long seen as a protein deficiency disease, manifests as a wasting and anorexic pathology only when certain bacteria are absent [107]. Asthma and allergies are also seen to be due to the absence of protective bacteria, which normally are present to be induce anti-inflammatory regulators [108,109]. In these studies, Hanski and colleagues [110] explicitly link environmental health, microbial diversity, and human health. Indeed, a new field of dysbiosis is now emerging, including not only infection, but also other conditions that may be caused by *deficient* or *aberrant* microbiomes. Here, the normal symbiotic relationships that maintain physiological or developmental continuity, have been abrogated. In recent years, science has traced these networks from associations to specific causal changes that can be tested. While many of these experiments have been performed in mice, the same pathways are known to be present in humans. These non-contagious diseases include asthma and allergy [111], kwashiorkor [107,112], obesity [113,114], diabetes [115], ulcerative colitis [116], depression [42,117], and Parkinson’s disease [118,119]. These “microbial deficiency diseases,” constitute a new and possibly important category of illness. This is not to say that dysbiosis is the only cause of these conditions, but that it is a public health concern that should be investigated.

An important conceptual barrier was recently crossed when the gut microbiomes of pregnant mice were demonstrated to be critical for the intrauterine development of the fetus. Short-chain fatty acids (such as butyrate and propionate) are products of the gut microbiome’s digestion of cellulose. As no mammalian genome contains genes for cellulose digestion, the breakdown of plant material is almost totally accomplished by enzymes produced by microbes. Kimura and colleagues [120] demonstrated that propionic acid, derived from the breakdown of fiber by maternal gut microbes, was critical for the normal development of the insulin-producing pancreatic beta cells, the sympathetic neurons that project to the heart, and the gut enteroendocrine cells. Without the microbe-derived propionic acid, the adult offspring developed a metabolic syndrome characterized by glucose intolerance, obesity, and insulin resistance. Since diet can control the prevalence of microbes, the holobiont model can explain the mechanism whereby eating low-fiber, high-calorie diets during pregnancy predisposes offspring to have metabolic syndrome later in life [121].

## 2. Mutualistic Symbiosis as a Public Health Issue 

### 2.1. Bodily Survival Depends on Symbionts

#### 2.1.1. The Body as a Multi-Species Consortium

To understand the importance of a symbiotic approach to public health, one has to first appreciate the new biological notion of the human body. Each of us is a functional entity that includes our zygote-derived cells as well as hundreds of species of microbes. The body is both an organism and a biome containing several ecosystems [28,76,101]. Once the amnion breaks, and the fetus passes through the birth canal, the newborn becomes colonized by their mother’s bacteria [87]. Furthermore, mothers’ milk contains a special set of nutrients to promote the survival and growth of those bacteria that are important for symbiosis [122,123,124]. These symbiotic bacteria will produce short-chain fatty acids and sphingolipids necessary for intestinal peristalsis and homeostasis, peptidoglycans necessary for normal neuron function, lipopolysaccharides necessary for the actions of the immune system, the tripeptides necessary for cardiac physiology, and the digestive enzymes necessary to metabolize plants [28]. Remarkably, a third of the small metabolites in the blood are produced or induced by bacteria [125]. Nearly all of our peripheral serotonin is induced by gut microbes, where it regulates the maturation of the enteric nervous system and regulates peristalsis [83,126].

Even more remarkable is that such critical symbioses are not only present within humans. Rather, the *development of most* organisms appears to be predicated on interactions between hosts and their symbionts. As mentioned in the above Section 1, without microbial symbionts, mice do not form their gut capillaries, their gut-associated lymphoid tissues, their T- and B- cell repertoires, or the proper synaptic connections in their guts and brains. Moreover, no vertebrate contains genes that make the enzymes necessary for digesting plant material such as cellulose, hemicellulose, and pectins [127]. These genes are provided by symbiotic microbes in our guts. In mammalian evolution, the entire family of ruminants is made evolutionarily possible by the ability of gut bacteria (acquired at birth) to build the rumen of the stomach and then to ferment grass and grains [128,129,130,131]. Thus, symbiosis is a paradigm-changing idea in physiology. We are no longer seen as being “individuals.” We are holobionts, and our anatomy, development, immunity, and physiology are intimately linked with that of our microbial components.

#### 2.1.2. Potential of Bacterial Displacement or Extinction to Cause Public Health Problems

The importance of the holobiont perspective for public health is that absences of particular microbes may cause dysbioses throughout a population. Martin Blaser and colleagues [6,132] have warned that we may need particular microbes for particular functions, and that our obsession with exterminating microbes may be inadvertently killing those bacteria that we need to survive. Their data indicate that microbes that used to be prevalent (those, for instance, in barnyards and horse stalls) are becoming rarer. If these microbes are necessary for normal organ, immune, or cognitive development, we will be impaired. Rhesus macaques that are bottle-fed, rather than breast-fed, acquire a different population of gut microbes, and this population is not as adequate to develop a functioning immune system that can repel opportunistic infections [133]. In zebrafish, a relatively rare species of bacteria is essential for permitting the expansion of insulin-producing pancreatic cells, thereby protecting these fish against diabetes [134]. 

This absence of specific bacteria (or their genes) may be crucially important for explaining the increases in allergies and asthma since World War I, and especially, after World War II. Throughout human history, we had constant exposure to barnyard microbes. It was only in the 20th century that they were displaced. The barnyard was not just an attribute of farms. The nineteenth century city, according to Raulff [135] (p. 36) “consisted of rows and rows of urban stables.” Mid 19th c. Boston had some 367 stables, each having around eight horses. In contemporary America, only 6% of Amish children, whose homes are often adjacent to their barns, have allergy and asthma. About 20% of the genetically similar Hutterites, whose farms are not located close to their homes, have allergies, roughly the same as the American population in general [136]. Similarly, Finnish studies have shown that proximity to the barn is a factor in combatting allergies. A recent study shows that children living in urban homes with barnyard bacteria have much less asthma and allergies than those children living in urban homes with urban bacteria [109]. Indeed, two of the bacterial types found in the “rural” homes and missing in “urban” homes were *Brevibacterium* and *Ruminococaceae*, bacteria found in horses and cattle. Although the severity of microbiome diversity loss might be most discernable in urban populations [137], the importance of soil microbiomes for the maintenance of healthy human intestinal microbiomes has recently been emphasized in studies [138] showing that even in rural areas, farming techniques have severely reduced soil microbiome biodiversity.

Bacterial displacement due to urban living and the absence of animals is only one of the ways that anthropogenic microbial displacement can affect public health. Caesarian sections disrupt one of the pathways of maternal kinship. Babies receive a protective set of symbiotic microbes from mothers when they pass through the birth canal. In caesarean sections, this transmission is abrogated. Babies delivered by C-section were found to be deprived of those microbes that otherwise colonize the infant gut. Instead, there were the hospital dwelling microbes that included a substantial number of opportunistic pathogens. Moreover, a substantial set of these microbes contained genes associated with antimicrobial resistance [139]. Not only were the species of microbes different, but so were their functions. The caesarean-delivered infants had less ability to mount immune responses to common antigens [140]. This may have strong public health implications concerning elective C-sections.

#### 2.1.3. In the Wings: Human Mental Health

The microbes of our gut are critical for “basic neurogenerative processes such as the formation of the blood-brain barrier, myelination, neurogenesis, and microglia maturation.” [141]. If this is indeed true, then could microbes also be critical for mental health? What if, in addition to protection against allergies and asthma, bacteria were protecting us against mental health conditions such as schizophrenia, bipolar disease, and autism? Several studies now indicate that gut microbes appear to be critical for normal brain development and behaviors in mice [82,142,143,144,145,146,147,148]. First, mice born from germ-free mothers and who are themselves without microbes have a syndrome that includes obsessive self-grooming and asocial behavior [149]. This behavior is possibly due to the failure of oxytocin-releasing signals from the vagus nerve, and it can be reversed by providing the germ-free mice with *Lactobacillus reuteri* or with microbes from normal mice or from normal humans [141,150]. Germ-free mice given microbes from autistic humans do not show improvement of their symptoms. Although human cognitive and affective behaviors cannot be extrapolated for those of mice, a pilot uncontrolled fecal transplant study in humans showed that after two years, the acquisition of normal bowel microbes by autism patients significantly improved their symptoms: from 83% severe autism to 17% severe autism [146]. Similar studies in mice and humans have shown that the gut microbiome may be critical in protecting humans from depression [42,151,152,153]. There is therefore reason to test the hypothesis that removal or depletion of normal environmental microbes may be responsible for the increasing percentage of the population diagnosed with cognitive dysfunction. While studies of the effects of microbes on mental health lag behind those studies of microbial involvement in physical health, the relationship of symbiotic microbes to cognitive and affective health and disorder is an area that cannot be ignored.

Public health must acknowledge that we are not monogenomic individuals. We are consortia of dozens of species per person, integrated together in a complex and dynamically changing network that forms who we are at any given moment. This network is altered by the food we eat, by the food our mother ate, the toxins and medications we are exposed to, and by our daily interactions with other holobionts. Our health depends upon other species, making the “One Health” perspective more than metaphor.

### 2.2. Environmental Survival Depends on Symbiosis

#### 2.2.1. Symbiosis Is a Defining Characteristic of Life

The symbiotic networks of the human holobiont are enmeshed in larger symbiotic networks that sustain the planet. Public health would be severely affected if any of the many life support systems on which we depend—including pollinators, soils, and bacteria—fail. Indeed, symbiosis is the signature of life on earth. The nitrogen in our soil and atmosphere is made available for protein synthesis by symbioses between rhizobacteria and legumes. The interactions of plant roots and mycorrhizal fungi are critical for plant growth, while endophytic fungi are often necessary to protect the plants against dessication [154,155,156]. The coral reef ecosystem is dependent on the symbiosis of algae and the ectoderm of corals, while the marine seagrass ecosystems are sustained by symbioses involving clams and their bacteria. Reef-building corals survive through the photosynthesis of their algal symbiont, which enters into the ectoderm of its host and transports over 90% of its photosynthetically derived carbon compounds to the host cells [157].

#### 2.2.2. Loss of Symbioses Affect Public Health through Environmental Degradation

However, these symbioses, the very symbioses that define the planet, are at risk. These are the analogues of the microbial displacement and extinction that affect human health. Although public health is mainly concerned about “human” public health, one readily finds that we cannot separate ourselves from our ecosystems socially, politically, economically, or biologically. Coral reefs, for instance, are thought to support 500 million people across 50 nations and contribute nearly a trillion dollars to the world’s economy [158]. The Great Barrier Reef, alone, brings 7 billion dollars annually to Australian commerce. Healthy coral reefs absorb over 95% of a wave’s energy, thus protecting the shoreline, preventing nearly a hundred million dollars’ worth of flood damage each year. However, the coral that form the critical structure of these reefs must be seen as a holobiont that exists only in a fragile symbiosis between the coral animal and single-celled zooxanthellae algae. The coral animal provides a sunlit, safe, and nutrient-containing environment for the algae; and the algae, living within the animal cells, provide the coral with the sugars it produces by photosynthesis. The coral holobiont can survive only when its symbionts are present to provide the food resources [157,159]. Under stress conditions such as high temperatures, the symbionts are expelled from the corals, leaving the corals “bleached” and undernourished. These corals usually die. As a result of global warming, massive bleaching events and coral die-offs have occurred [160,161]. We are writing this essay not only in the coronavirus pandemic of 2020 but in the Great Barrier Reef bleaching event of 2020 [162]. Over half the corals in the Great Barrier Reef have perished, and some entire reefs have collapsed. 

The mechanisms for the expulsion of the algal symbiont from its coral host are under investigation, and it appears to be a mutual breakdown of the symbiotic relationship [163]. One hypothesis is that heat disrupts the photosynthetic apparatus of the algae, causing them to produce dangerous hydrogen peroxide radicals. The coral cells defend themselves by expelling the algae or destroying them. Another hypothesis is that warmer temperatures permit algae to get the organic nitrogen that allows them to metabolize their sugars without needing the coral, thereby forcing the coral to rely on their own meager carbon reserves [164,165].

In addition to anthropogenic heating, humans are also affecting symbiosis through domestication. Mycorrhizal symbiosis is critical to plant nutrition and, therefore, a necessity for sustainable agriculture. However, artificial fertilization of soil diminishes the mycorrhizal fungi and root symbiosis. Martin-Robles and colleagues [166] have linked the loss of symbiotic colonization with plant domestication. Indeed, failure to colonize is common, making domesticated strains addicted to artificial fertilization [167,168]. Moreover, the lack of myccorhizal fungus may make the domesticated plants more susceptible to pathogenic fungi [169].

We are integrated into these webs, where our nutrition, oxygen, and environmental temperature depend on global symbioses, and microbes are at the base of each of them. The Anthropocene has put these relationships in peril. As Deborah Bird Rose [170] wrote, “Relationships unravel, mutualities falter, dependence becomes a peril rather than a blessing, and whole worlds of knowledge and practice diminish. We are looking at worlds of loss that are much greater than the species extinction numbers suggest.” 

#### 2.2.3. In the Wings: New Dangers from New Symbioses

The vectors of disease are following the sun and following airplane and sea lanes. Wastewater, tourism, and trade are circulating microorganisms around the world in a scale never before seen [171]. Moreover, global warming is predicted to introduce new microbes from melting permafrost as well as bringing many insect-borne diseases (Dengue fever, malaria, Lyme disease etc.) into new regions [69]. Here, the vector spreads a pre-existing symbiont. These will undoubtedly cause major public health concerns.

However, another mechanism of disease can be predicted: When organisms reach new lands, they are capable of finding new symbiotic partners. There is a new anthropogenic mingling going on. As an example, consider the red turpentine beetle, *Dendroctonus valens*, a minor pest species that routinely infects pine trees that have been damaged by weather or fire. Like other bark beetles, it is covered by fungi. These fungi digest tree bark, allowing the beetle to have a home and mate. The fungus associated with *D. valens* is usually *Leptographium procerum.* However, this beetle was introduced from the Pacific Northwest of America to Shanxi Province of China in the 1980s. In China, it met other fungi, which are much more potent at digesting wood than the American fungi [172,173]. These newly acquired fungi can degrade a major host defensive chemical [174]. As a result, over ten million pine trees have been killed by this fungus in China. American officials are worried about a “boomerang effect” [175]. The version of the beetle with its Chinese fungi may have been re-imported into the USA. However, the public health services of the various states that might be affected claim they do not have the revenue to test whether this is so.

Organisms are holobionts, and public health must recognize the webs of symbioses uniting different species of organisms into a collective “individual” and uniting these different individual teams into complex ecosystems.

## 3. AMR—Antibiotic Resistant Microbes as an Anthropocene Concern

### 3.1. Microbes as Parasitic Symbiosis

Symbiosis takes two major forms—mutualism (cooperative) and parasitism (pathogenic). The emergence of antibiotic drug resistance is the Anthropocene effect on parasitic symbiosis. Just as anthropogenic changes in the environment have changed the populations of microbes involved in mutualistic interactions with humans, so other anthropogenic changes have increased the prevalence and virulence of parasitic microbes.

Until the early 20 century, the leading cause of death, world over, was infectious disease. Crucial to turning this around were sanitation of water, and the discovery of antibiotics. Since their discovery in the 1920s, antibiotics have become the key tool against infections caused by microbes, used across different forms of medicine. By this definition, microbes are understood as pathogenic and parasitic, dangerous, dirty, and damaging the host that they reside in. Bodies are seen to be ‘at war’ against harmful outside invaders and entire disciplines have been hinged on this notion—immunology, clinical medicine, and public health just to mention a few. Antibiotics have been the miracle weapon that have been used to tackle the looming threats of bacteria and have been said to have developed contemporary medicine to be the success story that it is today [24].

Antibiotics have magnificent power to alleviate symptoms and ensure sterile conditions; they play central roles in basic surgeries, cancer, cesarean birth, and in treating basic infections. They are prescribed against infections by doctors, nurses, pharmacists, dentists, and traditional healers, depending on the contexts, all across the world. There are very few communities left that have not incorporated the use of antibiotics into their basic methods of healing, and research on those communities is tapping to their ‘untouched microbiomes’ microbiome as an ‘oasis’ [176,177,178,179]. Literature about antibiotic prescription describes how requests for antibiotics reside on all sides of the patient–health care practitioner dyad: patients say that health care practitioners hand out antibiotics liberally and health care practitioners argue that patients demand them [180,181,182]. In addition, antibiotics are bought over the counter from pharmacies, and informal markets [183]. Antibiotic use is a matter of concern as excessive or unregulated use of antibiotics is connected to the development of drug resistance and while there are few new antibiotics in the pipeline, there is a need to ensure the utility of existing ones [184,185].

Antibiotic use patterns offer insights into how central antibiotics are to public health, as well as the specific practices and contexts that rely on the use of antibiotics. Understanding these dynamics also illuminates the effects of pathogenic thinking as well as the myriad ways in which reliance on antibiotics would need to change in order to make space for a holobiont practice of public health.

Global statistics about antibiotic use show differences between countries that often follow the guidelines of health system efficiency and general national income. Since the 2000s, antibiotic use in low- and middle-income countries has considerably increased, while in high-income countries, particularly with those that rely on public rather than private health care, antibiotic use has been reduced. India, Pakistan and China are among those countries where use has increased most [186], while data is unavailable in most African countries [187,188]. That said, despite the reduction in the high-income countries, antibiotic use in many European countries and the US is still considerably higher per capita than across many African nations [186,188].

The increase of antibiotic use in low-income countries underscores the utility of antibiotics within lagging health care systems and/or in places where people cannot afford health care. Especially in countries where health care access is precarious due to lack of access, poverty, or poorly operating health systems, antibiotics have come to play a central role in how short-term health goals are achieved. For example, work by Denyer Willis and Chandler [189] shows how antibiotics function as a ‘quick fix’ for well-being. This fix operates on multiple domains: to ensure productivity of humans, animals and crops; hygiene in settings of minimized resources marked by lack of infrastructures; and good health in landscapes scarred by political and economic violence. In short, antibiotic use has come to stand for development and well-being.

While use of antibiotics has played a crucial role in helping to increase life expectancy, implementing invasive surgical procedures, and stand in for health care systems where they are otherwise unavailable, the use of antibiotics has accelerated embodied and ecological havoc. A narrow characterization of microbes solely as parasitic and pathogenic enemies rather than as needed and helpful partners contributes to excessive use of antibiotics for humans and animals, where microbes ‘refuse’ to remain contained in bodies but shift their form by evolving resistance to antibiotics. The heroic narrative of antibiotics is beginning to crumble as microbes push back. 

### 3.2. Antimicrobial Resistance and Plantationocene

Mass scale attempts to eradicate bacteria with antibiotics in humans and animals has led to increase of Antimicrobial Resistance (AMR), making it a quintessential Anthropocene problem. Indeed, the mass scale of antibiotic production, beginning in the 1940s, “quickly became infrastructural to the production of many other things at scale: more health, more meat, more fruit, more surgery, less death, more fertility, in everything from in vitro embryos cultured in antibiotics to fish farming. The scale of production is also the scale of resistance” [24]. The higher-than-expected levels of AMR put Western medicine in its current form—where antibiotics play central roles—at risk. With antimicrobial resistance, global health literature continues to frame microbes as a threat, now an incurable threat. The most comprehensive report about AMR and its future impacts, the so-called O’Neill Report commissioned by the UK government and the Wellcome Trust, indicated that 7 million people will die due to complications associated with AMR [190].

This report has evoked a flurry of research efforts, systemic interventions, stewardship programmes, and funding to tackle AMR. Health risks for humans have been extensively documented, with resistance spreading owing to both excessive use of antibiotics for human consumption and the use of antibiotics as part of animal feeding and in husbandry. A key route by which AMR spreads is via environmental bacteria that serve as vectors for the resistant genes—lateral gene transfer—which is seen to become a problem when otherwise benign environmental bacteria contribute to the spread of resistance in pathogens [24,191,192]. Robinson et al. state that this otherwise ‘natural’ quality of environmental bacteria is exacerbated, for example, by the influx of antibiotic residues from human and animal faeces, and run-offs from hospitals and pharmaceutical manufacturing [193,194].

A global comparison of socio-economic determinants correlated with AMR prevalence offers insights into the crucial roles that developmental and social inequalities play in Anthropocene ecology. Factors predicting high AMR rates are not antibiotic consumption, but, rather, differential access to sanitation, education, and public investment in health care services, as well as the level of corruption in society [195]. The focus, therefore, cannot be simply on clinical bodies, but must broaden to encompass environments including *animals and infrastructures* on the one hand, and *social practices and power* on the other.

We posit that the notion of *Plantationocene* captures this complexity that transcends the human–more-than-human bodily boundary while taking power structures into consideration. As defined above, the plantationocene constitutes the coercive labor structures and extractive and hierarchical management of planetary resources to feed an ever-growing population [17,18]. The plantationocene acts here both an analytical and a descriptive term. Analytically, plantationocene points to transnational circulations of goods, domination and dominion of people over other people and people over nature, hegemonic colonial legacies, systematisation of farming. Haraway et al. [196] point to the historical origins of the term and how relocations of the substances of living and dying around the Earth as a necessary prerequisite to their extraction. The logic of the plantation system makes it more efficient to destroy the local labor and import labor from elsewhere. The plantation system is built on the relocation and control of any generative unit, whether plant, animal, microbe, or person [196]. 

Indeed, plantations were the result of one of the most catastrophic public health events in world history—the Columbia Exchange. A major part of this exchange resulted in the elimination of a majority (perhaps 90%) of indigenous American people by the microbes—*Rubeola*, *Variola*, *Influenza*, *Rubulavirus*, *Rickettsia*, *Salmonella* and *Bordetella*—brought across the Atlantic Ocean by the European settlers. The great migration of people and crops took place to bring workers to areas whose native populations had perished, especially in the Caribbean, where the death rate of indigenous people was probably close to 99% [197,198,199]. Intensive labour was needed to produce crops in North and South America, and the ‘workers’ at plantations were slaves shipped from West and Central Africa—now sites that have the least infrastructure to surveil and control AMR, but have the most troubling evidence of AMR prevalence [187,200]. These were also sites of resource extraction as well as subjected to structural adjustments in the 80s by the World Trade Organisation to privatize health care and social welfare, resulting in poor health care and sanitation infrastructures and overall poverty that now are known to be key factors for the development of AMR. 

The industrial agriculture of the Plantationocene may also contribute to the spread of drug resistant microbes. The recent increase in resistant fungicides such as *Candida auris,* that has caused tremendous concern among health practitioners and ecologists alike, is a resistant yeast that has contaminated entire hospitals [201,202]. Its spread has environmental vectors—resistance has developed in connection to the use of fungicides in monocropping [203]. Environmental and agricultural practices are thus directly connected to public health concerns.

AMR with plantationocene underscores that public health needs to re-think its relationships with bacteria and antibiotics—it cannot bracket out environmental extraction, socio-economic injustices and the on-going need for health systems strengthening as factors that create the conditions for why excessive antibiotics are used that lead to antimicrobial resistance. AMR by this definition is not an exemplary threat by microbes as is framed in global public health but should be seen as a result of the modernist, eradication approach towards microbes that requires rethinking.

## 4. Conclusions

Health is a negotiation between microbes and hosts. Holobiont public health would do well to recognize both the parasitic and the mutualistic branches of symbiosis [204] It would also recognize the two major changes in our scientific knowledge of microbial evolution that have occurred in this century: (1) organisms are holobionts composed of several species, wherein microbes help maintain healthy physiology and resilience; and (2) bacteria can pass genes through horizontal genetic transmission, thereby facilitating the rapid spread of antibiotic resistance through numerous bacterial species.

Symbionts must be seen as partners and respected as agents with their own agendas. Three recent examples of holobiont “management” for public health should be mentioned in this regard. The first concerns the public health against mosquito-transmitted diseases such as dengue, Zika, and chikungunya by using *Wolbachia* bacterium to infect *Aedes egypti* mosquitos. *Wolbachia* infects numerous insects, but not these species of mosquitos. However, *Wolbachia* can become a symbiont in these insects, preventing the acquisition or replication of viruses inside their cells. Scientists have been able to get *Wolbachia* to grow inside *Aedes* cells, and *Wolbachia*-infected mosquitoes have been released into the wild. Where this has happened, there has been significant drops (up to 76%) in reported cases of the vector-transmitted diseases [204,205,206].

The second holobiont-informed type of public health involves seeking alternatives to antibiotics and partnering with microbes capable of keeping pathogens in check. If symbionts help protect hosts from pathogenic bacterial infections, then symbiotic microbes would be a good place to start looking for new antibiotics. This is especially true of antibiotics for Gram-negative bacteria. The antibiotics currently in use were developed in the 1960s, and several bacterial species have successfully been evolving resistance to them. Certain nematode worms are susceptible to the same types of Gram-negative bacteria as humans, so Imai and colleagues [207] sought out the antibiotics made by the symbiotic strains of bacteria found in the nematode guts. By screening chemicals made by these symbionts, they have isolated darobactin, a modified and crosslinked 7-amino acid peptide. This antibiotic acts by disrupting the cell envelope of the Gram-positive pathogens and is largely non-effective in destroying human gut commensals. The experiments further show that this new antibiotic is effective at protecting infected mice given potentially lethal infections of Gram-negative bacteria.

The third approach recognizes the importance of microbes to the life cycles of parasites and seeks to kill the parasite by killing its symbionts. This approach has worked in eliminating *Schistosoma mansoni*, a filariasis worm that has become resistant to the drugs traditionally used to kill these parasites. A newer treatment strategy has been to use antibiotics (such as doxycyline) against its symbiotic bacteria [208,209]. Once the antibiotic destroys the symbiont, the worms’ cells undergo apoptosis and the worms die [210]. A similar strategy is being considered to eradicate the plague locusts that are now devastating East Africa. Here, a locust-specific fungus might be sprayed on the juvenile locusts as they develop their wings. This fungus would grow inside the maturing insect and consume it from within [211].

We need to be in symbiosis with bacteria on a social, as well as on a corporal level. Like the body, we need to be able to distinguish mutualistic from pathogenic microbes and treat them differently. Humanity has been given notice. A paper by the Alliance of World Scientists [212] “puts humanity on notice that the impact of climate change will depend heavily on the responses of microorganisms which are essential for achieving an environmentally sustainable future.” Public health must take note that we humans are never independent of nature and, therefore, must be expanded to preserve environmental health as well as human and animal health.

Resilience to perturbations is increased by plasticity and the inputs of symbiotic microbes. Each human is a biome of many ecosystems. Asthma, allergies, and AMRs must not be considered the “revenge of nature.” Rather they are expected consequences of lower resilience to perturbations. Public health must see the life on this planet as biology now sees it—as a rich mixture of cooperative and antagonistic interactions, with our bodies in dynamic relations with its hundreds of microbial partners.
